# Cognitive behavioural therapy for insomnia (CBTi) as a treatment for tinnitus-related insomnia: protocol for a randomised controlled trial

**DOI:** 10.1186/s13063-019-3778-5

**Published:** 2019-12-02

**Authors:** E. Marks, C. Hallsworth, L. McKenna

**Affiliations:** 1 Department of Psychology, University of Bath, Claverton Down, Bath, BA7 2AY UK; 2 Royal National Throat Nose and Ear Hospital, 330 Gray’s Inn Road, London, WC1X 8DA UK; 3 Imperial College London, 535 Huxley Building, South Kensington Campus, London, UK

**Keywords:** Tinnitus, Insomnia, Cognitive behavioural therapy, CBT, Support groups

## Abstract

**Background:**

A significant proportion of patients with chronic tinnitus report clinical levels of sleep disturbance (insomnia). Despite the significant health and functioning implications of this, no rigorous trials have investigated treatments that target tinnitus-related insomnia. This is the first randomised controlled trial evaluating Cognitive Behavioural Therapy for insomnia (CBTi) in tinnitus compared with other psychological treatments.

**Methods/design:**

The study will test the efficacy of group CBTi as a treatment for tinnitus-related insomnia in a single-centre randomised controlled trial. Participants will be 102 patients with chronic, clinically significant tinnitus and insomnia in the absence of organic sleep disorders. Participants will be randomised to one of three intervention arms: six sessions of CBTi or six sessions of sleep support group or two sessions of audiologically based care. The primary outcomes will be changes in sleep as measured on the Insomnia Severity Index and key outcomes on a 2-week sleep diary (sleep efficiency and total sleep time). Outcomes will be collected 3, 10, 14 and 34 weeks post-randomisation. Secondary measures include sleep quality, sleep beliefs, tinnitus severity, psychological distress and quality of life. A sub-sample of participants will provide two weeks of actigraphy data at the same time points. Data on satisfaction and treatment experience will be collected at 10 and 34 weeks post-randomisation from all participants.

**Discussion:**

Findings from the study will be submitted to a peer-reviewed journal. It is anticipated that findings may inform future clinical practice in the treatment of tinnitus-related insomnia.

**Trial registration:**

ClinicalTrials.gov, NCT03386123. Retrospectively registered on 29 December 2017.

## Background

Tinnitus represents a significant population burden, with distressing tinnitus experiences reported by 1–2% of the population [1]. When tinnitus is bothersome, it is associated with many challenges, including difficulties with concentration, auditory perception, emotional distress and sleep. One of the most common problems is sleep disturbance, reported by 50–70% of people with tinnitus [2]. Despite being so widespread, insomnia in tinnitus is poorly defined and understood [3]. A recent review indicated prevalence rates of insomnia in tinnitus as varying from 10% to 80%, with most studies reporting rates above 40% [4]. However, diverse assessments have been used to define ‘insomnia’, and only one used full diagnostic criteria (reporting a rate of 27%).

Clearly, sleep disturbance is not inevitable in severe tinnitus, and many patients sleep well despite disabling tinnitus. Some reports, however, have suggested that tinnitus severity is associated with poorer sleep [5, 6] and lower quality of life. Yet, intervention studies to investigate whether insomnia-related tinnitus could benefit from more specialist intervention remain to be done.

This is of interest, given that there is evidence of similarity between tinnitus-related insomnia and primary insomnia. Cross-sectional studies comparing tinnitus patients with healthy control subjects using polysomnography have found that tinnitus is associated with increased sleep onset latency (SOL), as well as waking after sleep onset, decreased sleep efficiency (SE) and total sleep time (TST) [7]. Studies comparing patients with tinnitus-related insomnia with patients reporting primary insomnia show little significant difference between the groups on either objective measures of brain activity or subjective measures from sleep diaries and questionnaires, including daytime fatigue, mood and concentration [7, 8].

Stressful life events, associated autonomic hyperarousal, changes in serotonin and depressed mood have been identified as precipitants and maintaining factors in both tinnitus and insomnia [9, 10]. The cognitive models of tinnitus [11] and insomnia [12] share significant similarities, including stress arousal, negative thoughts about the symptoms, hypervigilance and monitoring for threatening symptoms, and safety-seeking behaviours. In patients with tinnitus, subjective measures of sleep quality and insomnia-related fears are elevated compared with healthy control subjects [13], suggesting typical insomnia-related cognitive behavioural processes (although direct comparisons with patients with insomnia are yet to be tested). Patients tend to attribute sleep problems to the noise of tinnitus. This attribution is difficult to validate, however, because both symptoms can be triggered by stress and illness, and tinnitus can cause stress and worry, further compounding insomnia. Overall, such findings suggest that cognitive behavioural treatments that can target primary insomnia could also apply to tinnitus-related insomnia [8].

This suggestion is yet to be tested because no clinical trials have targeted tinnitus-related insomnia. Sleep problems are not reliably measured or reported, and clinical insomnia is not identified within research samples. Psychological interventions for tinnitus are effective in terms of improving distress and quality of life, with evidence for cognitive behavioural therapy (CBT), mindfulness-based cognitive therapy and acceptance and commitment therapy [14–16], but none specifically target sleep. A few studies have found that sleep improved as part of general biofeedback and CBT interventions [17–19]. However, most include participants with non-clinical insomnia or have used poor-quality outcome measures [2, 20, 21].

Standard audiology-based care (ABC) for tinnitus-related insomnia involves one or two sessions with an audiologist and provides a mix of psychoeducation, advice to use external sound to partially mask tinnitus and advice about sleep hygiene (good habits regarding sleep/bedroom). A bedside sound generator is often provided, offering a choice of soothing sounds (e.g., wind, rain, waves, white noise). Various supporting literature may be given, such as information leaflets from the national tinnitus charity, the British Tinnitus Association. As described, reviews of tinnitus management rarely identify sleep as an outcome; as a result, there is no clear evidence regarding whether this ‘standard’ care for tinnitus provided by audiology services is in fact effective in managing insomnia. Thus the tinnitus literature offers minimal information about the nature and management of tinnitus-related insomnia, and studies are urgently required to improve understanding of this debilitating condition.

There are good reasons to suppose that specialised cognitive behavioural therapy for insomnia (CBTi) might help in this context. CBTi is the treatment of choice for primary insomnia, showing medium to large effect sizes [22, 23]. More importantly, it is also an effective treatment for insomnia secondary to other conditions such as pain, depression and cancer [23–29]. Tang and colleagues [26, 29] argued that secondary insomnia must be targeted directly using an approach such as CBTi and that sleep problems will not resolve alone, even if pain is successfully treated. They also noted that sleep deprivation can reduce pain tolerance. Because tinnitus shares some similarities with chronic pain [30], it is possible that similar processes apply to tinnitus-related insomnia. In chronic pain, the intervention for insomnia assumes that, although pain may trigger insomnia, the sleep problems are maintained and exacerbated by the development of behavioural habits around sleep. It is likely that this is also the case in chronic tinnitus. The reported benefits of CBTi in chronic pain settings led us to believe that it might also benefit patients with tinnitus-related insomnia. This is as yet not properly tested, but a recent clinical evaluation of group CBTi for tinnitus-related insomnia demonstrated significant improvements in sleep, tinnitus and distress [31].

### Defining insomnia

The insomnia literature offers the most useful definitions [24, 29], measures [24, 32, 33] and criteria for improvement [32, 34, 35], so far absent from the tinnitus literature. This will guide the definitions, measures and inclusion criteria used in this study.

### Rationale

Because tinnitus-related insomnia is common and associated with severe distress, it is important to test the most effective treatment. As yet, evidence for standard audiologically based care (ABC) for tinnitus-related insomnia does not exist, and whilst evidence for CBTi in a variety of health conditions is strong, it has not been tested in tinnitus. Robust testing will require a randomised controlled design. Because treatment intensity is different (CBTi involves six sessions, ABC rarely more than two) a third treatment arm involving six sessions of a supportive sleep group (SSG) will balance the CBTi contact time. Because tinnitus and insomnia are chronic conditions, a follow-up period of at least 6 months is required.

### Research aims and hypotheses

#### Primary objective

The primary objective is to assess the relative efficacy of CBTi compared with ABC for tinnitus-related insomnia from pre- to post-treatment and at 1- and 6-month follow-up time points.

Hypothesis 1A: CBTi will lead to a significantly greater reduction in insomnia than ABC from pre- to post-treatment and at follow-up, as indicated by mean changes on the Insomnia Severity Index (ISI).

Hypothesis 1B: CBTi will lead to a significantly greater reduction in insomnia than ABC from pre- to post-treatment and at follow-up, as indicated by mean changes on SE and TST, measured by a 2-week daily sleep diary.

#### Secondary objectives

Hypothesis 2A: A greater proportion of patients receiving CBTi will show a reliable clinical change in insomnia (> 6-point reduction on the ISI) compared with ABC and SSG.

Hypothesis 2B: CBTi will lead to a significantly greater reduction in insomnia than SSG from pre- to post-treatment and at follow-up, as indicated by mean changes on the ISI, SE and TST.

Hypothesis 2C: ABC will lead to a significantly greater reduction in insomnia than SSG from pre- to post-treatment and at follow-up, as indicated by mean changes on the ISI, SE and TST.

Hypothesis 2D: Compared with both ABC and SSG, CBTi will lead to significantly greater mean changes in measures of tinnitus-related distress, tinnitus catastrophising, psychological distress, anxiety, depression, quality of life and functioning from pre- to post-treatment and at follow-up.

Hypothesis 2E: A greater proportion of patients receiving CBTi will show a reliable change on the measure of tinnitus distress (> 11 points on the Tinnitus Questionnaire) compared with ABC and SSG.

Hypothesis 2F: Participants receiving CBTi will show a greater reduction in dysfunctional beliefs about sleep than those receiving ABC or SSG, and this reduction will be associated with greater improvements in insomnia.

Hypothesis 2G: There will be no significant differences between the three groups in terms of changes in subjective tinnitus loudness after treatment.

The study will monitor safety (incidence of adverse reactions), acceptability and patient satisfaction with each treatment. Adverse events will be solicited in the feedback form and may arise spontaneously. If reported, adverse events will be recorded and included in the final report. Participants will be offered psychological support outside of the trial, or the general practitioner (GP) will be contacted as appropriate.

## Methods/design

This randomised controlled trial will assess the effectiveness of CBTi for tinnitus-related insomnia using three independent groups and repeated measures. All three groups will be assessed at four time points post-randomisation: 3 weeks (pre-treatment), 10 weeks (post-treatment), 14 weeks (1-month follow-up) and 34 weeks (6-month follow-up). All interventions will take place within a single centre, a UK specialist ear, nose and throat hospital.

### Participants

Participants will be adults with chronic distressing tinnitus and related insomnia. They will be randomly allocated to receive CBTi, ABC or SSG.

### Inclusion and exclusion criteria

Participants are eligible if they report:

1. Presence of insomnia for a minimum of 3 months (scoring 15 or more on the ISI);
2. At least moderately distressing tinnitus for at least 6 months (scoring 8 or more on the Mini-Tinnitus Questionnaire [Mini-TQ]);
3. Sleep problems directly related to tinnitus;
4. Absence of organic sleep disorders (as assessed by a sleep disorder screening questionnaire);
5. Tinnitus has been fully assessed by a doctor/audiology specialist;
6. Aged between 18 and 70 years;
7. English proficiency and hearing level allow for participation in a group; and
8. Willing and able to provide informed consent to take part in a sleep-focused group treatment.

Participants will be excluded if they report:

1. Current, comorbid, severe physical or mental illness;
2. Active risk of harm to self or others;
3. Current substance dependence;
4. Current/planned pregnancy or breastfeeding; and
5. Medical investigations into sleep or tinnitus incomplete.

Participants taking hypnotic or psychotropic medication at the point of consent will be asked to keep this stable for the duration of the trial and not to engage in additional psychotherapy.

### Recruitment

The intention is to recruit 102 participants with distressing tinnitus and related insomnia from June 2017 to April 2019, which had been completed at the time of the proof-review of this article (November 2019). Participants will be identified from usual referral routes to the psychology service, where they will be informed of the trial as one option for care. To achieve adequate enrolment, the study will be advertised online via tinnitus charities, in print and web-based media outlets, across the hospital, and through regular briefings of local clinicians.

### Procedures

Participants responding to advertisements will complete basic eligibility screening with a clinical psychologist or research assistant using standardised measures of insomnia (ISI), tinnitus severity (Mini-TQ) and potential organic sleep disorders. If organic sleep disorder is indicated, a consultant in sleep medicine will manage queries, and onward referral will be advised as appropriate. If eligible, the GP will be informed and will have 3 weeks to ‘opt out’ of further care. Eligible individuals will be invited to attend a 1-hour full eligibility assessment with a clinical psychologist. Participants who are referred to the study by a clinician will complete eligibility criteria and the full assessment in the same appointment. If full eligibility is met, full informed consent will be collected by the clinical psychologist conducting the interview. The participants will receive sleep diary training and opt into Actiwatch (Philips Respironics, Murrysville, PA, USA) random allocation (one per group). They will be placed on a waiting list for randomisation, which will occur 3 weeks prior to intervention commencement. If eligibility is not met, alternative treatment or onward referral will be offered.

### Randomisation and blinding

Treatment will be group-based, with group sizes including six participants, on average. Treatment will be delivered in cohorts, with three groups, one for each arm of the study, running in parallel. Randomisation will be done using a customised computer algorithm for each cohort separately. The study requires individual randomisation with stratification for gender. Within each cohort, stratification will be used to ensure groups are comparable in gender composition. Because group size will be small per cohort, it was decided that stratification for age was not appropriate to run per cohort. Instead, age differences will be checked post-randomisation per group to ensure there are no significant group differences in age in each cohort.

Randomisation of consented participants will take place 3 weeks prior to the commencement of intervention. Age, gender and participant number will be provided to an independent party, who will use a computer-based random number sequence generation to allocate group. Allocation information will be returned to the research assistant, who will then notify participants of their group allocation (group 1, 2 or 3). Participants will only be informed of the treatment associated with their group number when they arrive at the first session. It is not possible to blind participants or therapists to allocation; however, participants will be blind to the content of the other therapies. Statistical analysis will be conducted by an independent statistician who will be blind to group (i.e., provided with group number, not group type).

### Intervention

All interventions will take place in the same place, on the same day, at different times. The same two clinical psychologists will conduct all treatments together. They both have significant experience, skills and knowledge of working with chronic tinnitus, insomnia, group therapy, supportive interventions and CBTi. Treatment sessions will be 120 minutes in duration, and follow-up sessions will be 90 minutes in duration. There are three intervention options:

1. **Audiology-based care (ABC):** ABC will be based on the best care currently available to patients with tinnitus and insomnia in the United Kingdom. There is no set standard, so this group has been designed to capture what is currently seen as best advice for people with tinnitus and insomnia. The group has been designed to be as effective as possible, because the intervention is being delivered by two clinical psychologists experienced in working in an audiological setting and in a supportive group setting. Intervention includes information and psychoeducation about tinnitus, sleep and relaxation. A bedside sound generator will be on loan for 3 months. Therapeutic contact is limited to two sessions 8 weeks apart. Supporting literature (leaflets from the British Tinnitus Association on sleep, tinnitus and relaxation) will be provided.
2. **Cognitive behavioural therapy for insomnia (CBTi):** CBTi will follow the UK standard treatment of primary insomnia, adapted for tinnitus. This multi-component treatment includes time-in-bed restriction, stimulus control, sleep hygiene, relaxation, paradoxical intention, cognitive restructuring, behavioural experiments and behavioural activity change. Additional discussions will focus on information, psychoeducation and advice about tinnitus management. Six sessions will occur over 8 weeks (four weekly sessions followed by two fortnightly sessions).
3. **Sleep support group (SSG):** This supportive intervention focuses on the benefits of clinical contact and a supportive milieu. It is an active condition controlling for the non-specific benefits of supportive group therapy. Sleep and tinnitus will be discussed from a personal perspective, focusing on personal stories and how participants cope. Participants will be encouraged to support each other. No expert audiological or psychological information will be provided, but therapists will facilitate discussion and adaptive group dynamics. Six sessions will follow the pattern of CBTi (four weekly sessions followed by two fortnightly sessions).

### Treatment fidelity

Treatment fidelity will be assessed by a clinical psychologist not involved in treatment. A random selection of treatment sessions will be recorded and assessed using a fidelity checklist. The two treating clinicians will discuss each session to assess fidelity independently of this, and breaches will be reported.

## Assessments and outcomes

### Timing of assessments

**Fig. 1:**
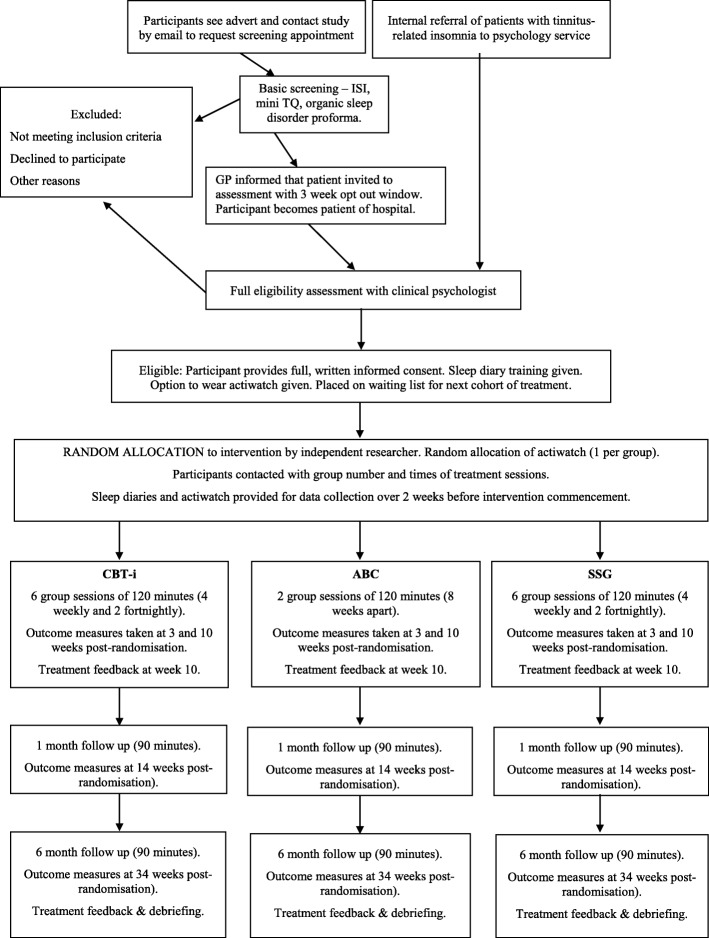
CONSORT flow diagram

Participants will be recruited from August 2017 to April 2019, which had been completed at the time of proof-review of this article (November 2019). Screening will be followed by full psychological assessment. Outcome measures will be reported at baseline (3 weeks post-randomisation) and at 10 weeks (post-treatment), 14 weeks (1 month follow-up) and 34 weeks (6-month follow-up) post-randomisation. Figure 1 shows the Consolidated Standards of Reporting Trials (CONSORT) flow diagram for the study.

### Managing loss to follow-up

The rationale for the repeated measures will be indicated to encourage follow-up. Completed measures will be checked so that missing items can be minimised. Participants who do not attend all sessions will be contacted and asked to complete and return outcome measures. Participants who request to end participation in the trial will be invited to an individual follow-up session and offered further treatment outside of the trial if required.

### Screening measures

Three screening measures will be used:

- Total score of the ISI patient version [36]. This seven-item self-report questionnaire assesses the severity, impact and characteristics of insomnia over the last 2 weeks. A 5-point Likert scale (from 0 = no problem to 4 = very severe problem) yields a total score ranging from 0 to 28, with scores of 15–21 reflecting moderate insomnia and higher scores reflecting severe insomnia. A minimum score of 15 (moderate insomnia) is required to meet eligibility criteria.
- Total score on the Mini-TQ [37], a psychometrically approved brief tool for effective assessment of tinnitus-related distress. A minimum score of 8 (moderate distress) is required to meet eligibility criteria.
- Sleep Disorder and Scoring Proforma, a clinical tool used routinely in the hospital, includes a list of common symptoms indicative of organic sleep disorders. Occurrence of symptoms will be discussed with a consultant in sleep medicine, and only those not requiring further sleep investigations will be included.

### Outcome measures

The primary outcome will be reduction of insomnia (ISI) and improvement in subjective sleep (diary) from 3 to 10 weeks, 14 weeks and 34 weeks post-randomisation. Daily sleep diaries will be completed by participants at home; questionnaire data will be completed independently in the treatment session and collected by the trial therapists.

- ISI has excellent internal consistency in patient samples (Cronbach’s α 0.91), is sensitive to treatment response and can show the clinical significance of change (whilst sleep diaries are limited to showing statistical significance) [33]. The ISI will be treated as a continuous variable, and groups will be compared on mean ISI change score from pre- to post-treatment and to follow-up. They will also be compared on the proportion of individuals showing change at the minimally important difference level, which is a reduction of at least 6 points [38]. Sleep diaries will allow calculation of the continuous variables SE (percentage of time in bed spent asleep), TST, and total wake time (TWT) over 2 weeks. Calculations will offer weekly mean averages and nightly variability measures.
- Secondary outcome measures will include additional measures of sleep, tinnitus, psychological distress and functioning, and all will be treated as continuous outcomes based on the mean or the total score (defined below). The Pittsburgh Sleep Quality Index [39] provides a global picture of severity of sleep problems by accounting for quantitative and qualitative dimensions. It has shown high sensitivity and specificity in distinguishing good and poor sleepers in samples of medical and psychiatric patients. It produces seven component scores summed to produce the global index score (0–21), with higher scores indicating lower quality and a score > 5 indicating ‘poor sleep’.
- The Dysfunctional Beliefs and Attitudes About Sleep Questionnaire–abbreviated version (DBAS-16) [40] is a self-report questionnaire designed to identify unhelpful sleep-related beliefs that are presumed to perpetuate insomnia and to assess changes in those beliefs that result from CBTi. There are 16 statements about sleep, to each of which participants are asked to agree/disagree on a 10-point Likert scale, and the mean score based on all 16 items is calculated. The DBAS-16 has good psychometric properties (α = 0.8).
- The Tinnitus Questionnaire (TQ) [41] will be used as our outcome measure for tinnitus distress. It is a self-report questionnaire with 41 items that contribute to a total score on five sub-scales (emotional disturbance, intrusiveness, auditory perceptual difficulties, sleep disturbance and somatic complaints). Items are scored 0–2, with total or sub-scale scores calculated [41]. There is high test-retest reliability (*r* = 0.94) and internal consistency (α = 0.93) [42]. In addition to comparison of change in total TQ score across groups, the TQ will be categorised to indicate the number of individuals who show reliable change from pre- to post-treatment and to 6-month follow-up. Reliable change on the TQ is indicated by a reduction of at least 11 points [16].
- The Tinnitus Catastrophizing Scale (TCS) [43] assesses negative cognitions about tinnitus on a 13-item scale, which has shown good internal consistency. The total score of the TCS will be analysed as a continuous variable.
- Subjective tinnitus loudness will be measured as a continuous variable using a visual analogue scale (VAS).
- Psychological distress will be assessed using the 34-item Clinical Outcomes in Routine Evaluation–Outcome Measure (CORE-OM) [44]. This pan-diagnostic measure of general psychological distress assesses well-being, symptoms, functioning and risk with 34 items rated on a 5-point scale (0 to 4). Internal reliability of the CORE-OM domains is appropriate (ranging from α > 0.75 to < 0.95). Convergent validation against other measures and clinician ratings is good [45]. Groups will be compared on change in the CORE-OM clinical score (calculated as the mean score, multiplied by 10). CORE-OM clinical score will also be used to categorise individuals as those who show reliable change from pre- to post-treatment and to 6-month follow-up (a reduction of 5 points or more) [16].
- The Patient Health Questionnaire-9 (PHQ9) will assess depressive symptoms on nine items rated on a 0 to 3 scale, where a score of 10 or more equates to clinically significant symptoms [46]. Groups will be compared on change in total score.
- The Generalized Anxiety Disorder Assessment-7 (GAD-7) will assess anxiety symptoms on seven items rated on a 0 to 3 scale, where a score of 8 or more demonstrates clinically significant symptoms [47]. Groups will be compared on change in total score.
- The Work and Social Adjustment Scale (WSAS) [48] measures general functioning in terms of impairments related to tinnitus measured on five items rated on a 0 to 8 scale, where a score of 10 or more indicates clinical significance. Groups will be compared on change in total score.
- The widely used EuroQoL (EQ-5D) [49] will measure health-related quality of life. Five questions assessing different dimensions (mobility, self-care, usual activities, pain/discomfort and anxiety/depression). The answers are converted into EQ-5D index and utility scores anchored at 0 for death and 1 for perfect health. A VAS reports on subjective health status from 0 (worst) to 100 (best). Groups will be compared on mean change in index score and VAS.
- Change in mean scores on subjective measures from 2-week sleep diaries (rated as 0–10) will be compared between groups, including nightly rated sleep quality, tinnitus annoyance (0 to 10) and daily rated daytime functioning.

### Additional measures

- Baseline demographic and medical information will be reported: age, gender, ethnicity, marital status, education level, tinnitus duration, hearing loss, sleep, other audiological problems, other health problems and previous treatments. Previous research has indicated that sleep can change with age, so age will be included in the final model as a covariate.
- Use of psychotropic and hypnotic medications will be collected at each time point. If participants commence such medication, their results will be excluded from analysis. We will report numbers of any such changes across all groups.
- Use of night-time sound enrichment, naps, caffeine and alcohol will be collected at each time point because the offer of sound generators and advice regarding sleep hygiene is a systematic difference between treatment groups. We will report proportions of participants using night-time sound enrichment across groups, and we will also report proportions of patients showing reductions in caffeine, alcohol and naps across groups.
- One participant per treatment arm per cohort will be randomly allocated an Actiwatch to wear during the same time period as sleep diary completion. This will provide an objective comparator for the subjective diary data. This measure is limited because there is insufficient hardware available to include all participants.

Following treatment, participants will indicate the usefulness and relevance of treatment on 11-point Likert scales (0 to 10). They will be asked to provide qualitative feedback regarding their experiences and views of treatment.

**Fig. 2:**
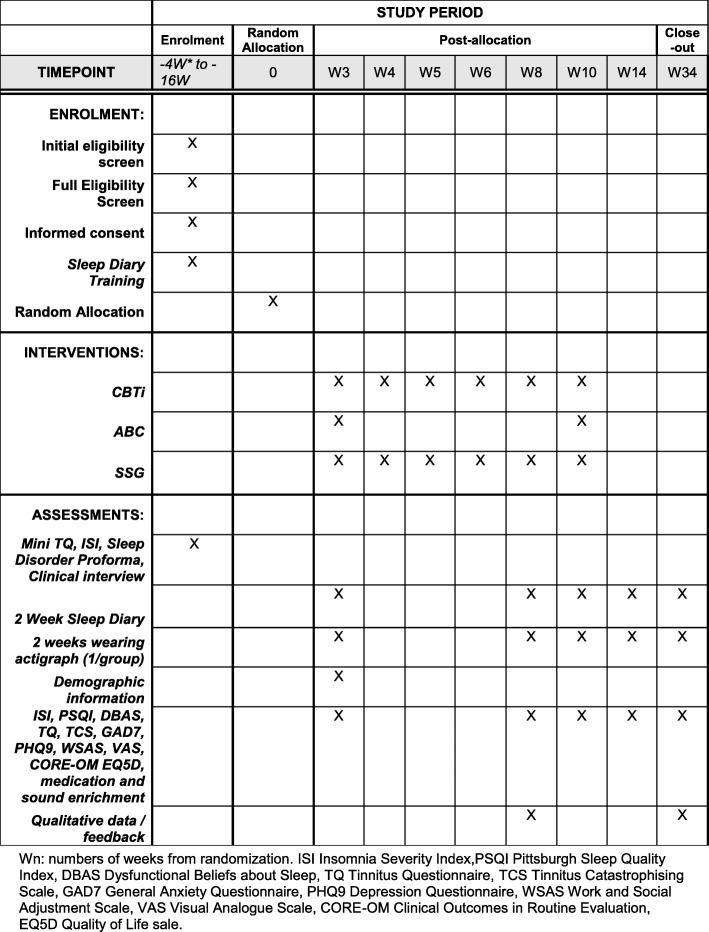
SPIRIT diagram

A diagrammatic representation of the trial process (enrolment, intervention and assessment) is shown in the Standard Protocol Items: Recommendations for Interventions (SPIRIT) diagram (Fig. 2). For the full SPIRIT checklist, please see supplementary material Additional file 1.

### Sample size

We calculated the sample size required to test the primary hypothesis on the basis of a recent meta-analysis of 14 randomised controlled trials comparing CBTi with control groups in the treatment of primary insomnia [22]. Here, between-group comparisons reported effect sizes on subjective sleep variables (Cohen’s *d*) as ranging from medium (*d* = 0.44) to large (*d* = 1.09) across a range of control groups (including no intervention, placebo control, waiting list, treatment as usual and information controls). Similarly, medium to large effect sizes were seen on self-rating measures. When looking at treating insomnia in the context of a chronic health condition such as pain, similar moderate to large effect sizes are reported [27]. These findings have led us to base a power analysis on an assumed effect size of 0.8.

Estimation of potential loss to follow-up was set at 10%, based on rates reported by Okajima et al. [22], as ranging from 0% to 33% and as 8% from a clinical evaluation of CBTi within a tinnitus clinic [31]. We assumed a cluster design with six patients per group, and we assumed a within-group correlation of 7%, estimated from a previous study [31]. Correlation between measures was estimated as 25% on the basis of a small pilot study. It is likely that the true correlation will be higher, so this estimate has a conservative effect on required sample size. Assuming a significance level of 5% and 80% power, the number of participants needed to detect a statistically significant difference between the CBTi and ABC groups on the primary measures of interest was 34 per group (102 across all three groups).

### Statistical analysis

The three groups will be compared at baseline on outcome measures and demographic information. Data will be reported as mean (SD) on continuous variables, (primary and secondary outcomes), and as percentage (number) for categorical data (demographic data, change in use of sound enrichment, medication, naps, caffeine, alcohol). A logistic model will be used to assess predictors of missing data and to examine all baseline characteristics and demographic variables.

The primary hypothesis to be tested is that CBTi will lead to a significantly greater reduction in insomnia in patients with tinnitus than usual care (ABC) from pre- to post-treatment and at follow-up, based on the primary outcomes of total ISI and 2-week average of SE and TST from a daily diary.

### Primary outcome analysis

The ISI and sleep diary data will be analysed using linear mixed models, accounting for repeated measures on participants and the cluster structure of the trial. Post hoc comparisons of independent groups will allow multiple comparisons by using adjusted *P* values. We will also conduct an analysis to indicate the number needed to treat based on reliable change in the ISI.

Analysis will be based on modified intention to treat (excluding those who contribute no data). For data missing at random, multiple imputation will be used. To minimise missing data, the research assistant will follow up on missing data after questionnaire completion.

### Secondary outcome analysis

As for the primary analysis, a linear mixed model will be used to investigate differences in outcome between the three groups at additional time points (1-month and 6-month follow-up) and on all secondary outcome measures described above. In addition, correlational analyses will be used as a quality control to check for the level of association between actigraphy and sleep diary data, including TST, TWT, SOL and SE.

Regression analyses will be conducted to assess whether changes in the primary outcome measure (ISI, TST, SE) are associated with changes in sleep beliefs (DBAS-16) score.

Satisfaction will be assessed via a single three-group analysis of variance on treatment usefulness and relevance. Written responses will be analysed using thematic analysis. Treatment adherence will be assessed on the basis of attendance rates. Categorical outcomes, including adverse events and non-adherence, will be recorded, reported and compared with Fisher’s exact test.

### Sensitivity and other planned analyses

Additional sensitivity analysis will be conducted as follows: outliers (analyses with and without outliers), non-compliance (a per-protocol analysis), baseline imbalance (analyses with and without adjustment for baseline characteristics, if imbalanced), and impact of distributional assumptions (analysis plan will assume a normal distribution for continuous outcomes, and this will be tested for goodness of fit in the analysis). In addition, sensitivity analyses will be conducted using other suitable distribution under further statistical advice.

## Ethics and dissemination

The study has been approved by the London – Camden and Kings Cross NHS Research Ethics Committee, and it is being supported by University College London Hospitals (UCLH) and sponsored by University College London (UCL). All treatments are based on best evidence and expected to benefit participants. All assessments and interventions are provided by highly trained clinical psychologists with expertise and knowledge in treating tinnitus and insomnia. All participants who have consented will be patients of the hospital, and safety will be managed in line with hospital protocol. Issues of safety arising at screening will be communicated to the GP and relevant professionals. Trial conduct will be audited by the research team. Because all treatments are well-known procedures and the trial cannot be blinded, no data management committee will be required.

Once completed, the study will be disseminated via publications in peer-reviewed journals, in presentations at relevant conferences and online through the British Tinnitus Association website. Any modifications to protocol will be communicated to the relevant research ethics committee, trial participants, ClincialTrials.gov, UCL and UCLH.

Data will be handled, stored and disposed of in accordance with applicable legal and regulatory requirements, including the UK Data Protection Act 1998 and the NHS Code of Confidentiality. Source data will be kept within the patients’ psychological records in a locked filing cabinet in a locked room. Electronic data (questionnaires or diaries provided in an electronic format) will be printed out and stored in the same way. For analysis, to ensure confidentiality, data will be fully anonymised, non-identifiable and collated on a spreadsheet. Participants will be given a unique, confidential trial identification number attached to their paper files, and this will be used for identification. Data will be accessible only to the research team and to regulatory authorities within UCL and UCLH. Currently there is no ethical approval to share these data more widely. Data will be input by a research assistant not involved in treatment. Data quality will be promoted with checking a sub-sample of input data and range checks for data values.

## Discussion

CBTi is a new intervention that may improve treatment for individuals who have insomnia related to their chronic tinnitus. A small, uncontrolled evaluation has indicated that CBTi may be an effective treatment [31], but this has not been compared with existing treatments. Therefore, in this trial, we will test CBTi against two common interventions available to patients with tinnitus.

This study has some limitations. Firstly, neither the practitioner nor the participants can be blinded to allocation. This is a common issue in psychological treatment trials because interventions cannot be delivered or received in a blinded way. This is somewhat mitigated by blinding participants to the content of the alternative treatments and only informing them of their allocation in their first treatment session and by ensuring that data analysis is conducted by a member of the team who is blinded to group.

The protocol adheres to SPIRIT 2013 [50]. The trial will be monitored for integrity to methods and scientific validity. The results will offer valuable data about the efficacy of psychological treatments for tinnitus-related insomnia.

## Supplementary information

**Additional file 1.** SPIRIT 2013 checklist: Recommended items to address in a clinical trial protocol and related documents*.

## Data Availability

The datasets that will be generated and/or analysed during the current study will be held by the research team within UCLH. They will not be publicly available, owing to the ethical agreement obtained for this study and to maintain individual privacy.
